# Thrombosed left ventricular pseudoaneurysm following myocardial infarction: a case report

**DOI:** 10.1186/s13256-021-02709-5

**Published:** 2021-05-03

**Authors:** Fatma Zouari, Rami Tlili, Fares Azaiez, Sofien Zayed, Rim Ben Romdhane, Jalel Ziadi, Youssef Ben Ameur

**Affiliations:** 1 Department of Cardiology, Mongi Slim University Hospital, La Marsa, Tunis, Tunisia; 2 Department of Cardiovascular Surgery, La Rabta University Hospital, Tunis, Tunisia

**Keywords:** Pseudoaneurysm, Myocardial infarction, Transthoracic echocardiography

## Abstract

**Background:**

Pseudoaneurysm of inferior wall of the left ventricle is an uncommon complication of myocardial infarction with high mortality.

**Case presentation:**

We report the case of a 63-year-old Tunisian man, diagnosed with a thrombosed left ventricular pseudoaneurysm and a pericardial effusion after 1 week of angina.

**Conclusions:**

Left ventricular pseudoaneurysm is a serious complication of myocardial infarction that has atypical presentations. Diagnosis is generally established by transthoracic echocardiography but confirmed by magnetic resonance imaging. Urgent surgery is the treatment choice given the risk of embolization and rupture.

**Supplementary Information:**

The online version contains supplementary material available at https://doi.org/10.1186/s13256-021-02709-5.

## Introduction

In ST-segment elevation myocardial infarction, time is muscle. Despite awareness campaigns and the introduction of primary percutaneous coronary intervention as the principal reperfusion strategy following acute ST-elevation myocardial infarction, patients are still presenting late, when complications are more frequent and prognosis is poorer.

The mechanical complications of myocardial infarction are the most formidable and are accompanied by high mortality. Left ventricular pseudoaneurysm is a serious complication that can lead to heart failure, arrhythmia, distal embolization, and rupture of the left ventricular free wall. It is often associated with a significant mitral regurgitation and/or interventricular communication. Cardiac ultrasonography is a simple and reproducible tool that allows early detection of these abnormalities.

## Case presentation

**Fig. 1:**
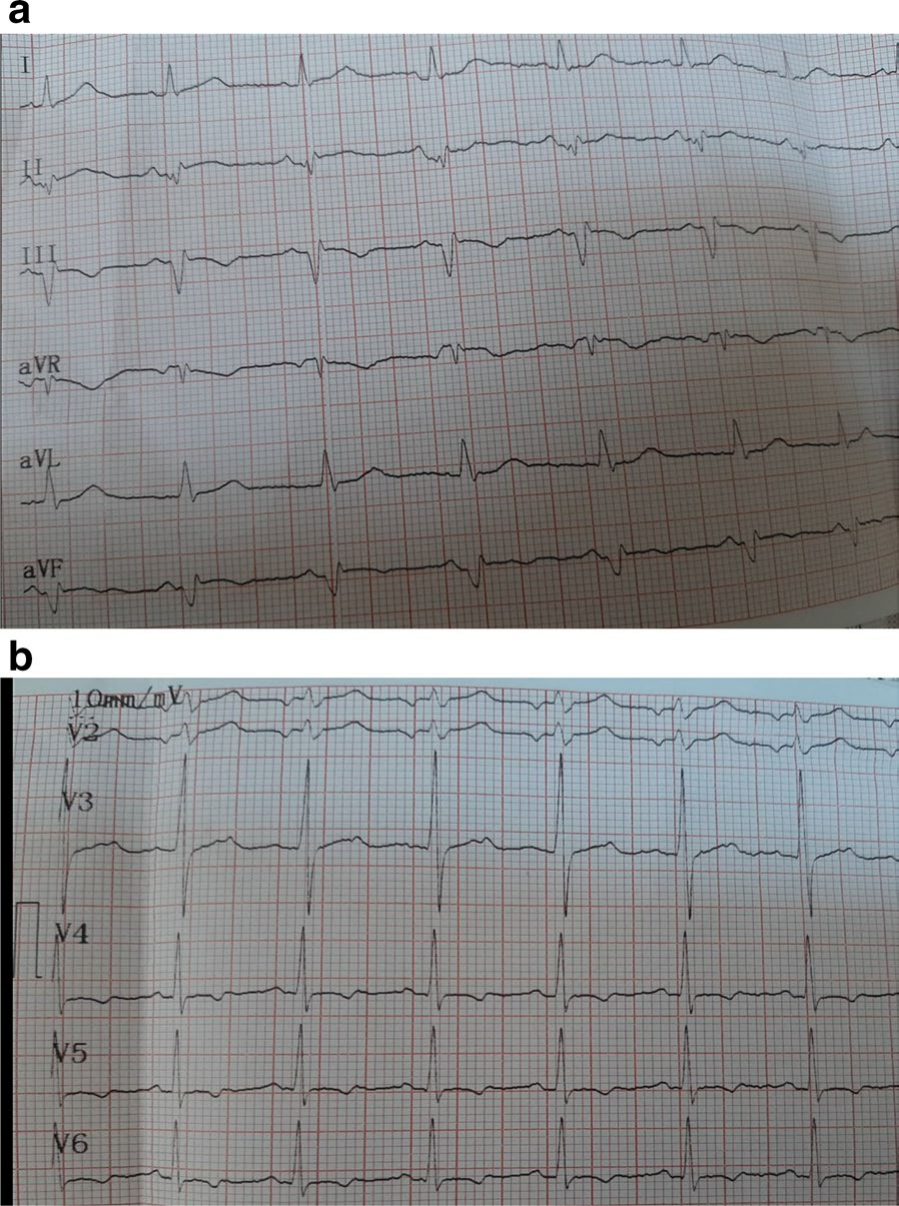
**a**, **b** Electrocardiogram at admission

**Fig. 2:**
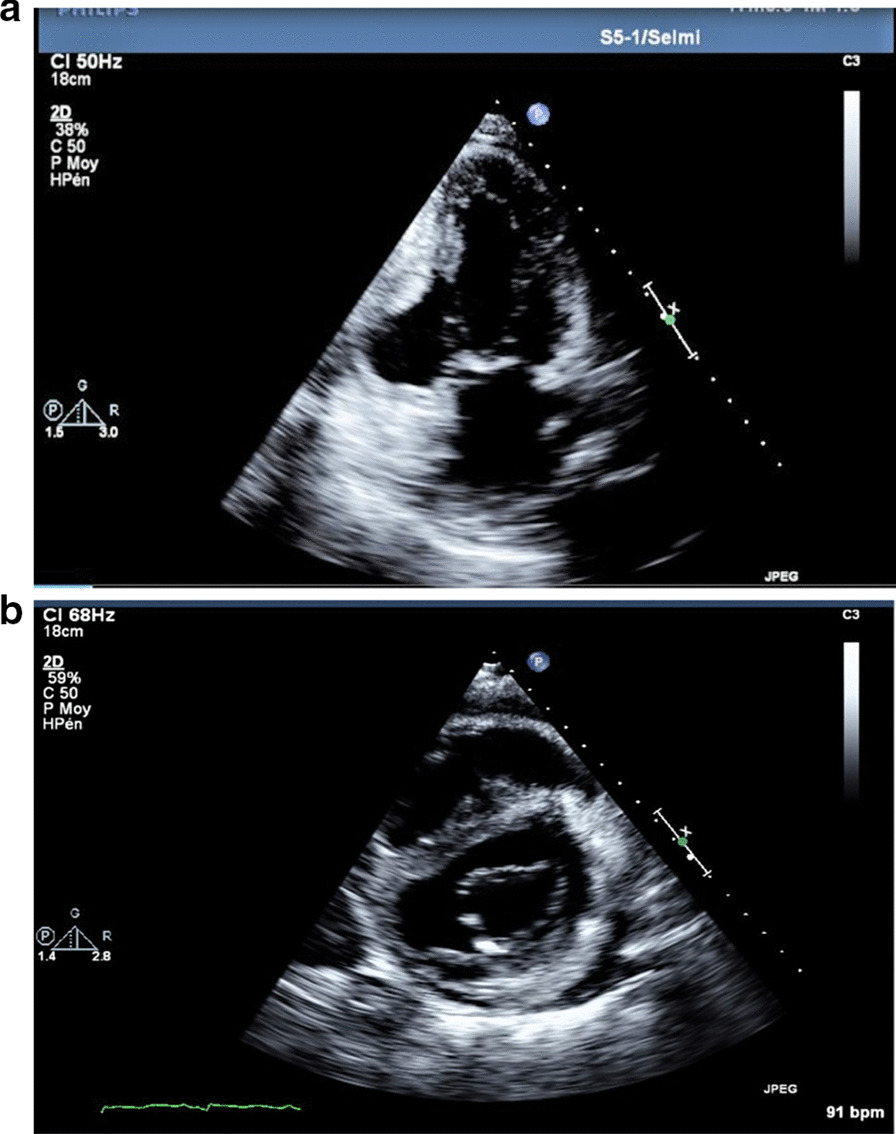
**a** Transthoracic echocardiogram from apical two-chamber view showing a large pseudoaneurysm from the inferior and inferolateral wall of the left ventricle. **b** Transthoracic echocardiogram from parasternal short axis view showing a large pseudoaneurysm from the inferior and inferolateral wall of the left ventricle

**Fig. 3:**
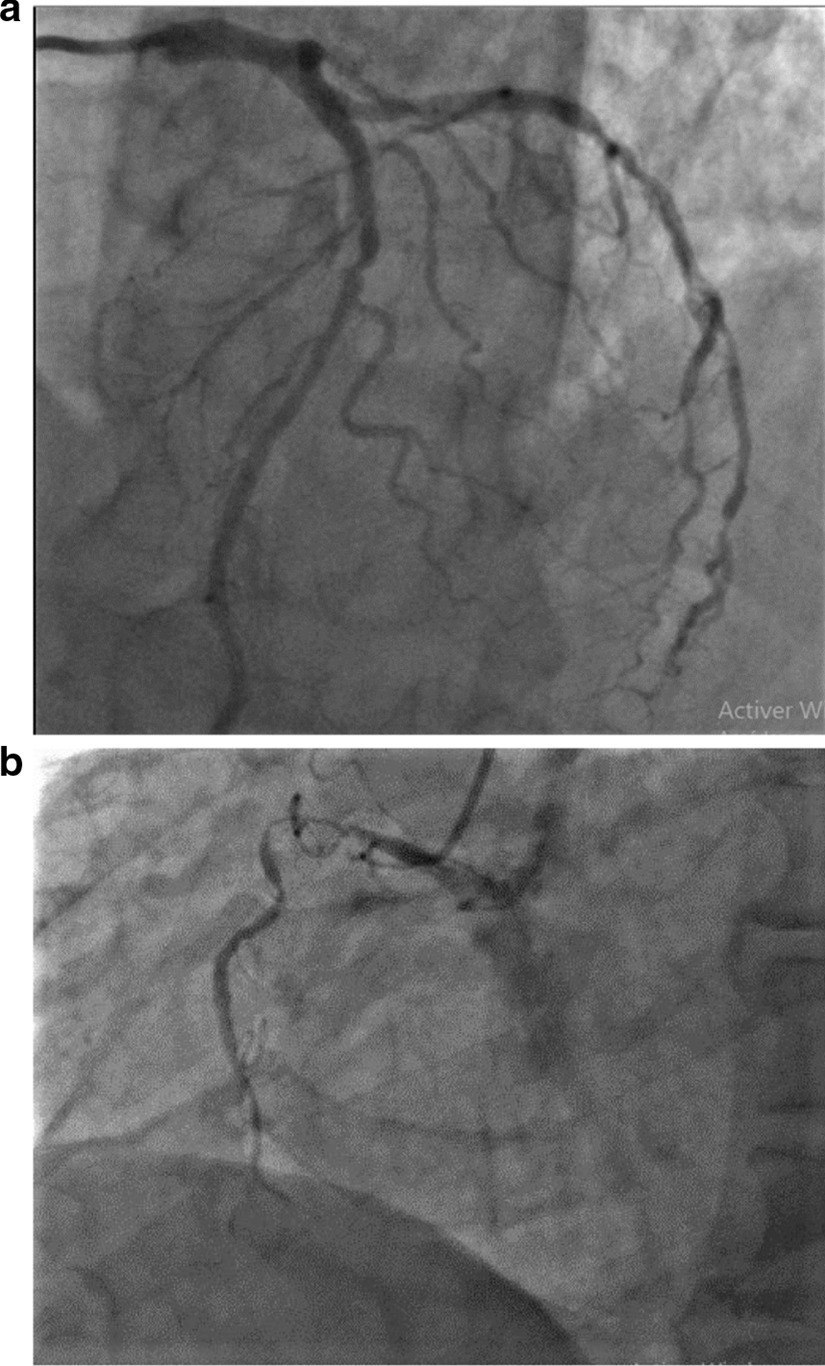
**a**, **b** Coronary angiography showing bitroncular lesions

**Fig. 4:**
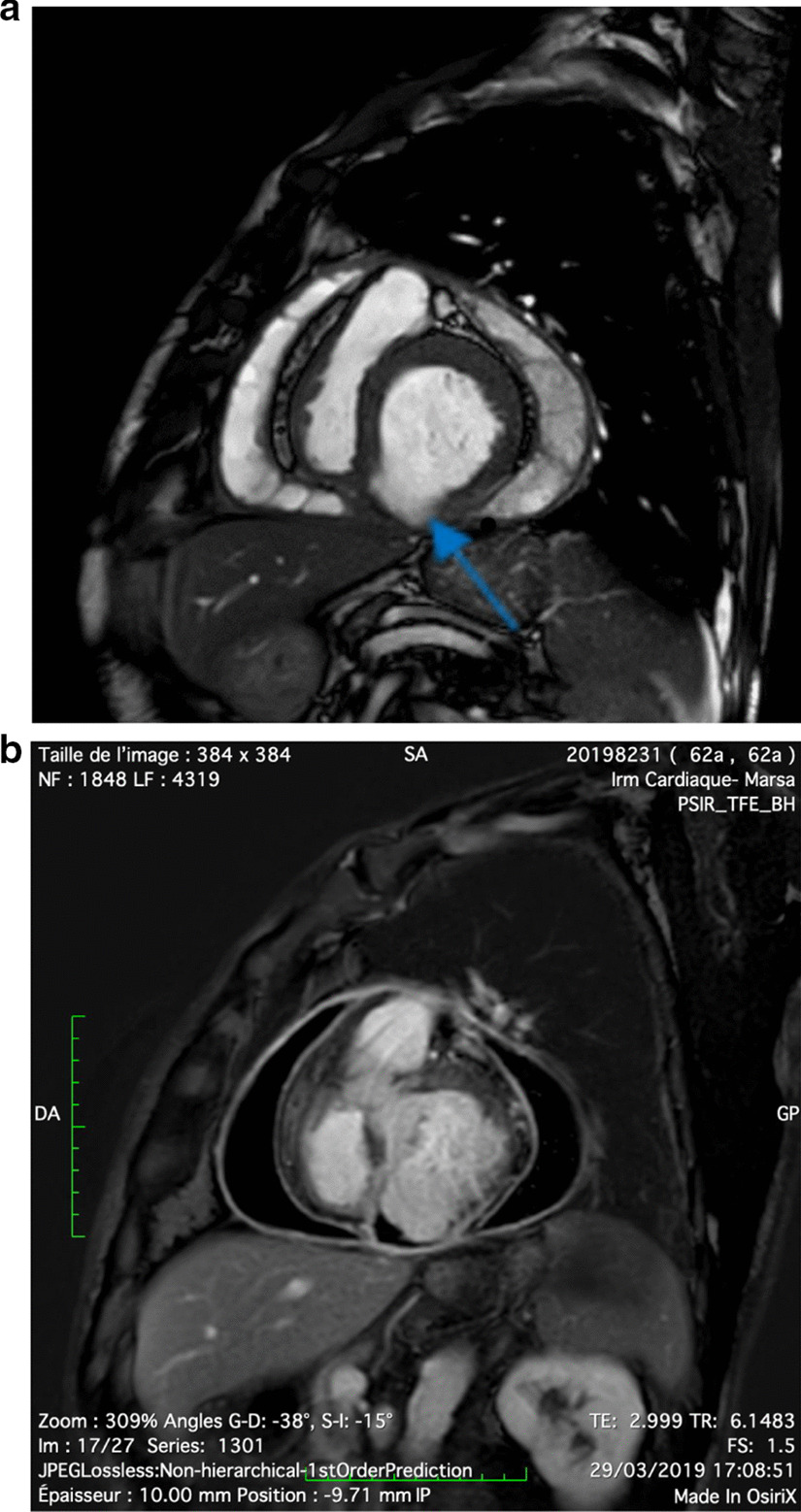
**a**, **b** Magnetic resonance imaging T2 sequence in axial section showing the false aneurysm of the inferior wall with a linear hyposignal opposite the endocardium corresponding to the thrombus (shown by the blue arrow) with huge hemopericardium

**Fig. 5:**
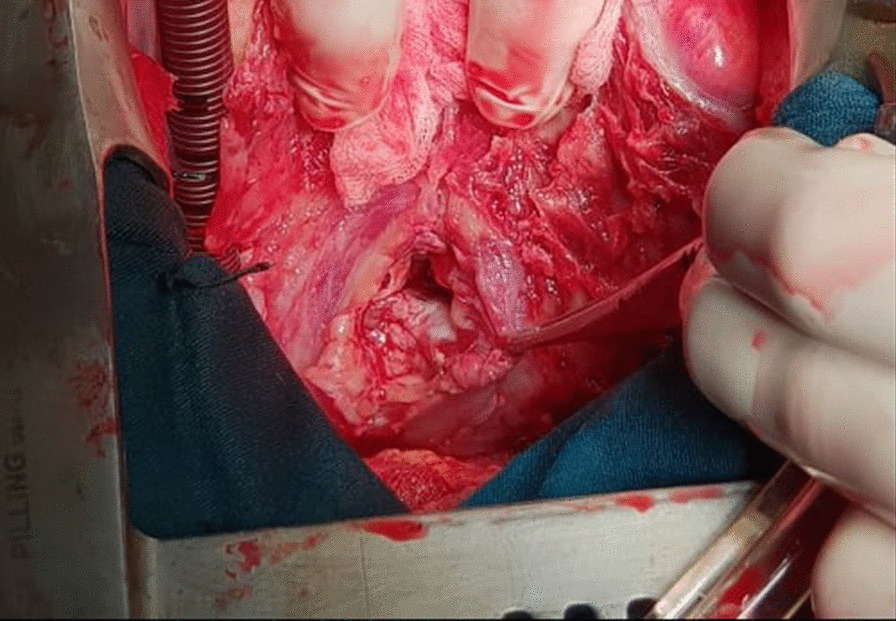
Intraoperative incidence showing rupture of the free wall of the left ventricle

**Fig. 6:**
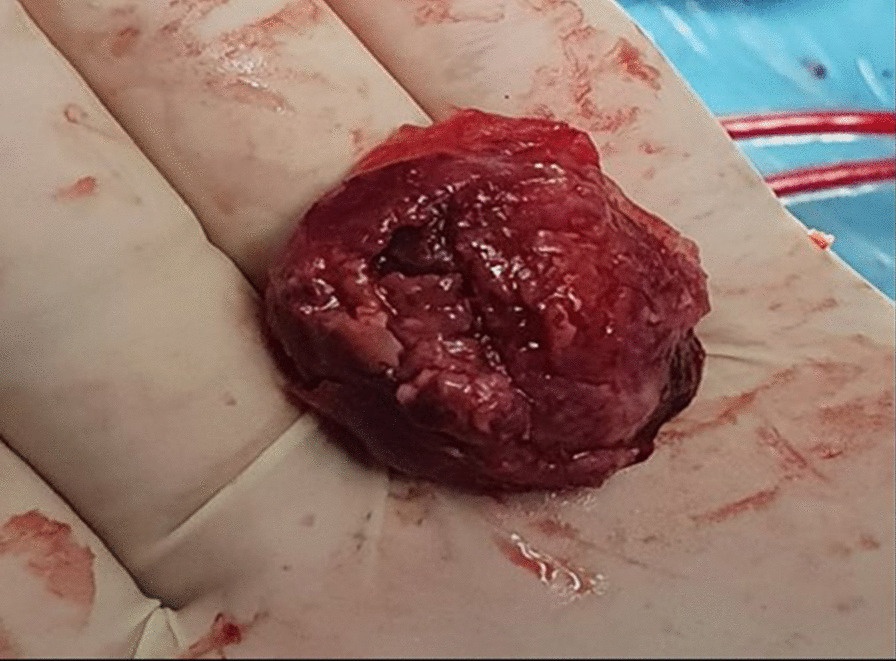
The fibrino-cruoric thrombus

**Fig. 7:**
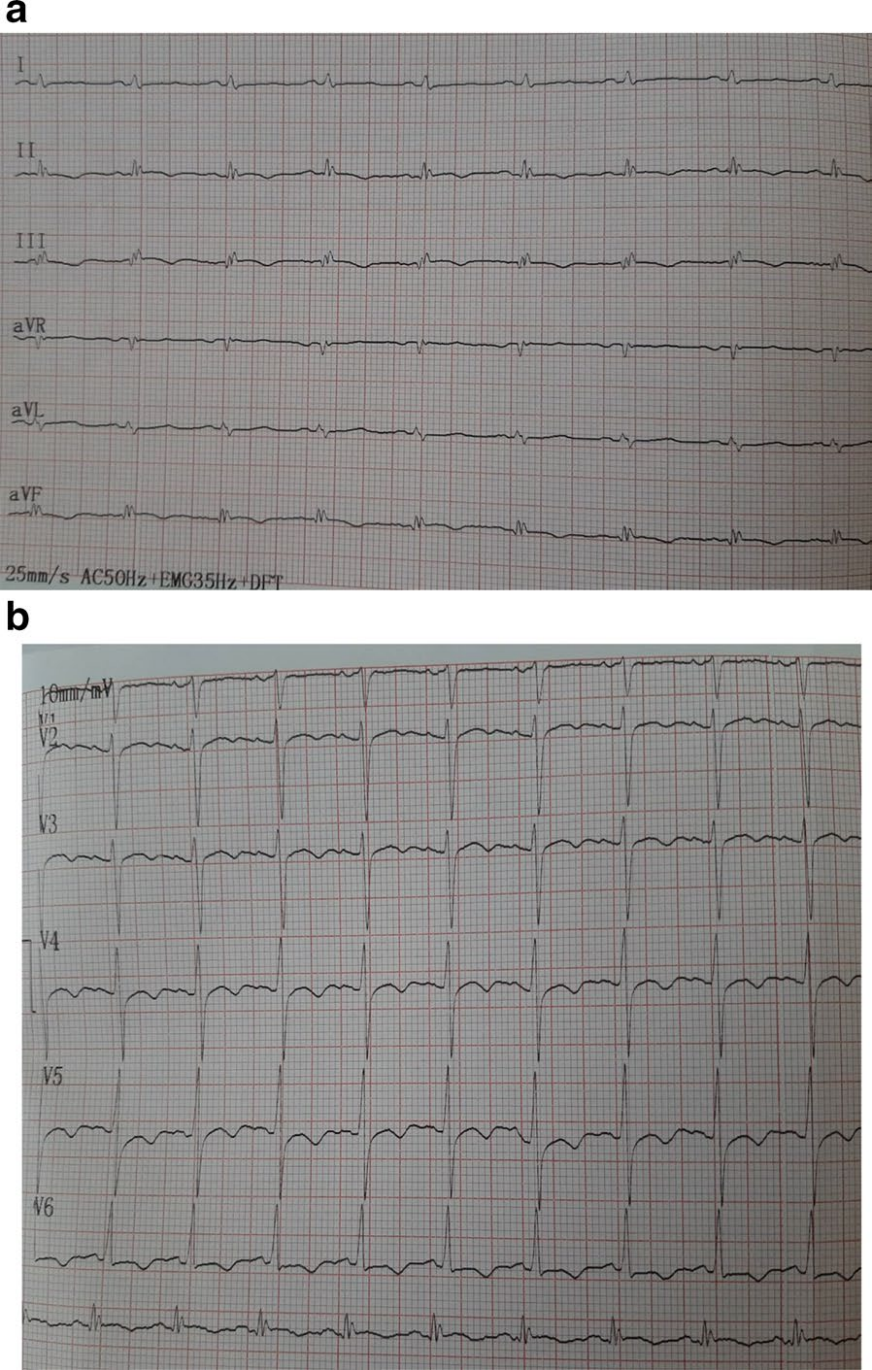
**a**, **b** Electrocardiogram after operation

**Fig. 8:**
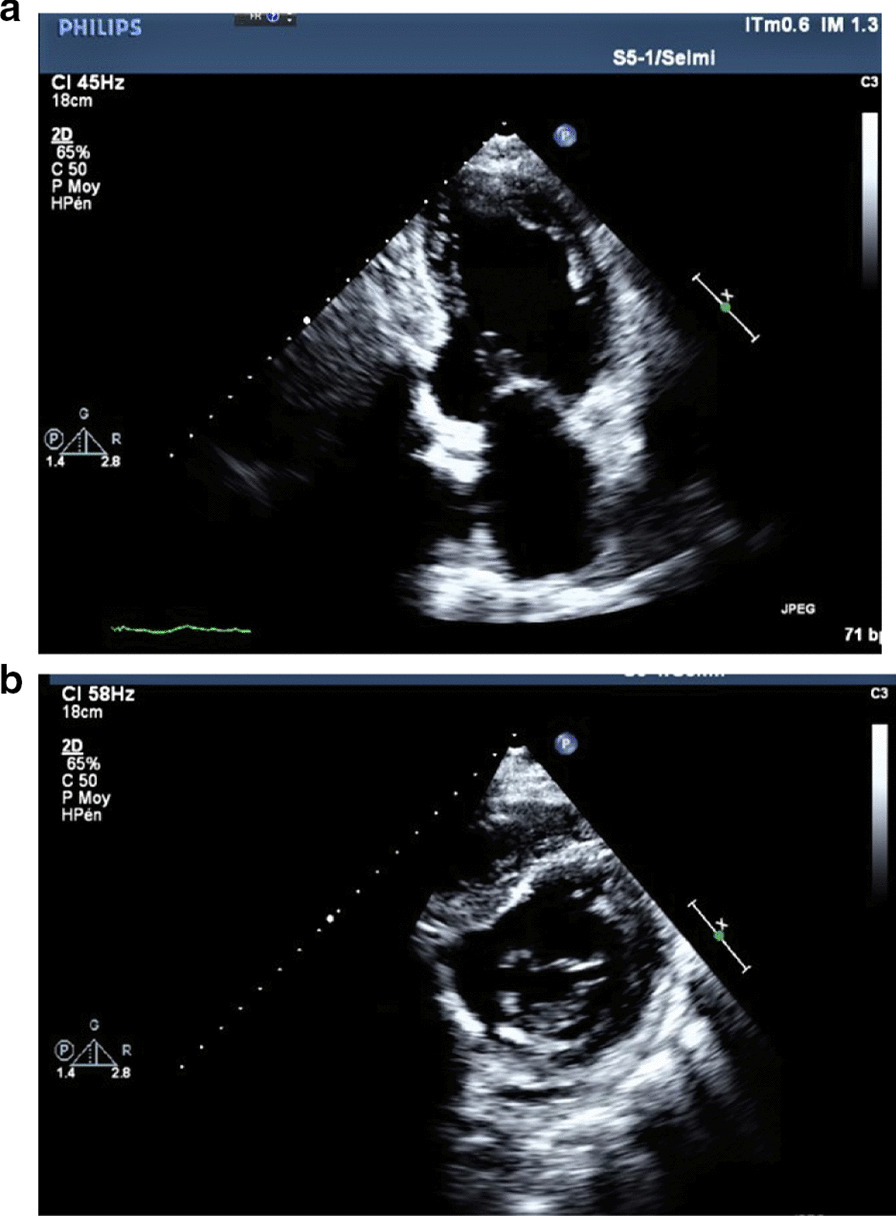
**a** Postoperative transthoracic echocardiogram from apical two-chamber view showing ventricle restoration. **b** Postoperative transthoracic echocardiogram from parasternal short view showing ventricle restoration

We report the case of a smoking, diabetic, hypertensive 63-year-old Tunisian man, who consulted the emergency department for pericardial syndrome. During the investigation, we learned that angina had been present since one week, associated with nausea, but ignored by the patient. On admission, his blood pressure was 110/74 mmHg. Physical examinations revealed mesocardiac systolic murmurs. Electrocardiography showed a regular sinus rhythm at 75 beats per minute and Q wave necrosis in the inferobasal derivations (Fig. 1a, b). Transthoracic echocardiography revealed a huge aneurysm (33 x 19 mm) in the basal segment of the inferior and inferolateral wall of the left ventricle and a reduced systolic function (35%) without significant mitral regurgitation (Fig. 2a, b) (Additional files 1, 2: video 1a, b). There was also a circumferential moderate pericardial effusion. Coronary angiography showed a long critical occlusion in the proximal segment of the right coronary artery and critical stenosis of the left anterior down to the mid segment (Fig. 3a, b). Magnetic resonance imaging (MRI) was performed after angiography, revealing non-viable myocardium in the territory of the right coronary artery complicated by an image of addition 51 mm long axis, to a wide neck (30 mm), partially thrombosed from the inferobasal wall of the left ventricle (LV), fused and inseparable from the pericardium, causing a false aneurysm. It also showed a huge and compressive hemopericardium (Fig. 4a, b). The patient was quickly taken to a cardiovascular surgery center. Intraoperative results confirmed the diagnosis of false aneurysm by the presence of a dehiscence of the lower wall of the LV measuring 30 mm in diameter (Fig. 5), clogged with a fibrino-cruoric thrombus (Fig. 6). It was decided to perform surgical aneurysmectomy with myocardial Dacron patch reconstruction and artery bypass grafting to the left anterior descending artery using the internal mammary artery. The culprit lesion (right coronary artery) was not treated surgically because of the non-viability in its territory. Postoperative course was uneventful, intraaortic balloon pump was removed on the 4th postoperative day (POD), the patient was extubated on the 5th POD, and was discharged on the 16th POD without complications (Figs. 7a, b, 8a, b) (Additional files 3, 4: video 2a, b). At his 18-month follow-up, the patient remained well with satisfactory exercise tolerance. Transthoracic echocardiography showed improvement in ejection fraction (from 35 to 45%) (Additional files 5, 6: video 3a, b).

## Discussion

Rupture of the left ventricle wall is a fatal complication of myocardial infarction, with a significant hospital mortality of up to 80% [1]. This complication is rare, and its incidence has decreased progressively and in parallel with the improvement of myocardial reperfusion strategies [2] and the early use of drugs, especially low-molecular-weight heparin and beta blockers [1]. Incidence has decreased from 4 to 0.2% [1]. Rupture of the anterior wall is more serious than rupture of the posterior wall because it is more likely to cause a hemopericardium, whereas rupture of the posterior wall often enables the generation of an inflammatory reaction of the posterior pericardium at the origin of pericardial adhesions and the development of a pseudoaneurysm [3]. Posterior pseudoaneurysms are the most frequent, accounting for 83% of false aneurysms [4]. Pseudoaneurysm is sometimes associated with other mechanical complications, such as significant mitral insufficiency (by reaching the posterior papillary muscle) [5] and interventricular communication [6]. These pseudoaneurysms may be asymptomatic (> 10%) [7] or present with nonspecific signs such as congestive heart failure, angina, rhythm disturbances, or thromboembolic events, which occur after a variable delay of the acute event, after an average of 50 days [8]. Thus, pseudoaneurysm is rarely diagnosed in the clinic. It is important to know how to distinguish between an aneurysm and a pseudoaneurysm because the therapeutic cares differ. Left ventricular angiography associated with coronary angiography was the reference examination to confirm the diagnosis of LV pseudoaneurysms and to assess the need for associated coronary bypass [7]. Transthoracic ultrasound is currently the reference. It provides important information regarding the anatomy and localization of the defect and the presence of a thrombus or associated valvulopathy. It can also assess left ventricular function and look for pericardial effusion. Transesophageal ultrasound appears to provide more information than transthoracic ultrasound, especially in terms of posterior pseudoaneurysms [9]. Meanwhile, the diagnosis is not always obvious. Zoffoli *et al.* [5] had therefore established certain ultrasound and angiographic criteria making it possible to differentiate between false and true aneurysms. MRI has 100% sensitivity and a good negative predictive value. It successfully identifies thrombi and delayed enhancement of pericardium [10]. The European Society of Cardiology recommends transthoracic ultrasound as the first-line examination to confirm pseudoaneurysms and suggests that MRI can complement the diagnosis by identifying the contained cardiac rupture and its anatomical features to guide surgical intervention [11]. In our patient, MRI provided us with additional information regarding the presence of thrombus and the viability of the myocardium of the affected territory. Urgent surgery is the best treatment given the risk of embolization and rupture of pseudoaneurysms, which is of the order of 30–45% [7]. It consists of an aneurysmectomy and patch closure. However, the risk of recurrence of these pseudoaneurysms still exists, and 5-year survival is only about 60% [8]. Some cases of percutaneous closure for patients at high surgical risk have been described with good results [12]. There are some who have even opted for conservative treatment [13].

## Conclusion

LV pseudoaneurysm is a rare and serious complication of myocardial infarction. It should be suspected in post-infarction patients who have unexplained clinical signs, especially dyspnea or angina, regardless of the time of onset after the infarction. Transthoracic ultrasound is an easy and accessible test to confirm the diagnosis. Surgery is the mainstay of treatment but has high mortality. Long-term survival is also low.

## Supplementary Information

**Additional file 1: Video 1a.** Transthoracic echocardiography from apical two-chamber view showing a large pseudoaneurysm from the inferior and inferolateral wall of the left ventricle.

**Additional file 2: Video 1b.** Transthoracic echocardiography from parasternal short axis view showing a large pseudoaneurysm from the inferior and inferolateral wall of the left ventricle and pericardial effusion.

**Additional file 3: Video 2a.** Postoperative transthoracic echocardiography from apical two-chamber view showing left ventricular remodeling.

**Additional file 4: Video 2b.** Postoperative transthoracic echocardiography from parasternal short axis view showing left ventricular remodeling.

**Additional file 5: Video 3a.** 18-month follow-up transthoracic echocardiography from apical two-chamber view.

**Additional file 6: Video 3b.** 18-month follow up transthoracic echocardiography from apical four-chamber view.

## Data Availability

The datasets used and/or analyzed during the current studies are available from the corresponding author on reasonable request.

## Abbreviations

LV: Left ventricle
MRI: Magnetic resonance imaging
POD: Postoperative day
